# Hidden scars: the impact of violence and the COVID-19 pandemic on children’s mental health

**DOI:** 10.1186/s13034-020-00340-8

**Published:** 2020-09-10

**Authors:** Najat Maalla M’jid

**Affiliations:** Special Representative of the UN Secretary-General on Violence against Children, United Nations Secretariat, 304 East 45th Street, 17th Floor, New York, NY 10017 USA

**Keywords:** Violence against children, Child rights, Mental health, Sustainable development goals, COVID-19 pandemic

## Abstract

More than 1 billion children are exposed to violence every year. The devastating immediate and long-term impact of violence on the mental health of children is well established. Despite commitments made by the international community to end violence against children and support their mental health, there has been a serious lack of investment and capacity to provide quality, rights-based, culturally appropriate mental health care globally. The arrival of the COVID-19 pandemic has magnified these challenges. This article outlines how the risk of children experiencing violence has increased and how the pandemic has weakened the capacity of child protection and mental health services to respond. The article argues for child protection, mental health and other core services to be prioritized during and after the pandemic. A failure to do so will undermine the international community’s ability to achieve the Sustainable Development Goals by 2030 and to fulfil its obligations under the UN Convention on the Rights of the Child.

We are in quarantine, and we can’t go out. The situation is very bad. People are experiencing anguish and desperation at home. (Natalia, age 16, Peru).

Coronavirus is affecting us very seriously. First of all, stress. We get depressed because of staying home. It affects me, [and I am feeling] not so much joy. I feel like I can’t stay in the house. (Alexandru, age 13, Romania).

I do not like this situation. We wanted to announce the end of Ebola, but now coronavirus is already coming. We stay at home, we no longer study, we no longer go to church, and we are unable to participate in meetings. And, despite the fact that isolation will help protect us from the virus, this will bring starvation that can still kill us too. (Anita, age 16, Democratic Republic of Congo) [1]

The UN Convention on the Rights of the Child guarantees the right of every child to freedom from violence and to the highest attainable standard of mental health. Similarly, the 2030 Agenda for Sustainable Development pledges to end all forms of violence and to promote mental health and well-being.

Yet more than 1 billion children—half of all children in the world—are exposed to violence every year [2]. They face violence in many forms and in many places, whether they are online or offline. A child can be the target of violence, can witness it directly, or be otherwise exposed to it.

The devastating immediate and long-term impact of violence on the mental health of children is well established. These consequences include depression, post-traumatic stress disorder, borderline personality disorder, anxiety, substance use disorders, sleep and eating disorders, and suicide. Exposure to childhood violence can increase a wide range of adult psychopathologies, including disorders that affect mood, anxiety levels and behaviour [3].

Despite commitments made by the international community, there has been a serious lack of investment and capacity to provide quality, rights-based, culturally appropriate mental health care globally, even though mental health is consistently identified by children themselves as a major concern [4]. Far too few children with mental health problems receive the right support at the right time.

The arrival of the COVID-19 pandemic has magnified these challenges. While the data we have so far are only indicative, the mitigation measures taken in response to COVID-19 have heightened the risk of children experiencing or being exposed to violence at home due to school closures, confinement measures, and added family stress related to job loss, isolation, and anxieties over health and finances [5]. The World Health Organization has also reported that children with disabilities, children in crowded settings and those who live and work on the streets are particularly vulnerable to violence and abuse [6].

The widespread use of online platforms to mitigate the impact of school closures on children’s education has exacerbated the problem of violence against children online, with children spending a greater proportion of unsupervised time on the internet.

In addition to the impact of the violence experienced by children during the pandemic, the stress and uncertainty associated with the outbreak potentially has significant negative effects on children’s mental wellbeing. In consultations undertaken directly with children, they have expressed that they feel more unsafe, insecure, scared, lonely and isolated [1].

Child abuse is less likely to be detected during the COVID-19 crisis, as child protection agencies have had to reduce monitoring to avoid spreading the virus, and teachers are less able to detect signs of ill treatment with schools closed. The disruption of protective services can have a particularly high toll on children who are already in a vulnerable situation, such as those living in humanitarian settings.

Moreover, the increase in people in need of mental health or psychosocial support during the pandemic has been compounded by the interruption to health services in many countries. Care systems have been affected by mental health staff being infected with the virus and the closing of face-to-face services. In many countries, community services have been unable to meet [6]. The result has been that many children with existing mental health conditions have not been able to receive the necessary care and support.

The pandemic will also have a negative impact in the longer term. The economic crisis will roll back development gains in tackling poverty and will increase children’s vulnerability to violence. The World Bank has estimated that up to 100 million more people may be forced into extreme poverty [7]. The International Labour Organization and UNICEF report that a one percentage point rise in poverty leads to at least a 0.7 per cent increase in child labour in certain countries, which will reverse the progress on decreasing child labour for the first time in twenty years [8].

Poverty is also a driver of child trafficking, sexual exploitation and recruitment of children into criminal gangs, as well as into armed groups and forces. Equally, poverty increases the risk of child marriage: the United Nations Population Fund projects that an additional 13 million child marriages may take place over the next 10 years [9].

The United Nations issued a joint Agenda for Action on Child Protection and COVID-19 to highlight the steps States need to take to ensure that children’s protection from violence is prioritized in the response to the pandemic [10]. It was guided by the call of the UN Secretary-General to ensure that what began as a health crisis does not evolve into a broader child-rights crisis.

Action on mental health is a crucial component of this Agenda for Action. It called for practical support to be provided to parents and caregivers, including how to talk about the pandemic with children, how to manage their own mental health and the mental health of their children, and tools to help support their children’s learning. It further urged States to ensure the continuity of child-centred services as a core element of the response to COVID-19, including mental health and child protection services. There have been important steps taken by States to achieve this: such initiatives should be evaluated and those found to be effective should be emulated and scaled up.

As the international community looks ahead to building back better after the COVID-19 pandemic, there is an opportunity for governments worldwide to reassess priorities. Mental health and child protection services must be recognized as life-saving and essential services—along with other health services, social protection and education—as part of an intersectoral and child rights-based response.

Advancing human development and reducing inequalities, especially for children, calls for a major investment. This includes actions targeting the drivers of violence against children. It should also encompass the modernization and scaling up of mental health services as an essential part of universal health coverage, emphasizing the development of community services and building the human resource capacity to deliver quality mental health and social care. As Special Representative of the UN Secretary-General on Violence against Children, I have called on States to deliver these changes, most recently in a report to the UN Human Rights Council [11]. In a forthcoming report on the harmful impact of violence on the mental health of children, I will highlight a range of cost-effective, evidence-based interventions that can be promoted to achieve this.

It is essential that children be part of the solution in the immediate and recovery phase of this pandemic. Even during this challenging time, children’s resilience, activism, and sense of solidarity are remarkable. With the use of digital technology, children around the world are providing peer-to-peer support to help ease the stress they experience, taking their activism online to share safety information among their peers and volunteering to provide support to other children in need.

The financial impact of the pandemic will undoubtedly affect the resources at States’ disposal to mitigate its effects. However, even where resources are constrained, there is always a choice. The cost to children and to society of not strengthening mental health and child protection systems in the recovery phase is simply too high to ignore.

We are at a crucial turning point in the lives of the generation of children who will be most affected by COVID-19. We must do all that we can to ensure they do not become the main victims of the pandemic, but instead build a better world where their rights are promoted and protected, and where no child is left behind. If we fail to do so, the promises made to children through the Convention on the Rights of the Child and the 2030 Agenda will not be kept.

## Data Availability

Not applicable.
